# The Physician’s Hand-Book of Practice for 1860

**Published:** 1859-11

**Authors:** 


**The Physician's Hand-Book of Practice for 1860.**

We have received, from the Publishers, W. A. Townsend & Co., 46 Walker street, New York, this little manual, and after a careful examination of its contents, have no hesitation in saying, that it is the most complete work of the kind hitherto published. The thanks of the profession are certainly due Drs. Elmer & Elsberg for their great care in getting up this little volume. It contains a diary, visiting list, a pocket account book, a classical list of remedial agents, and in a compass sufficiently small to be readily carried in the pocket. Price $1 25, post-paid, upon receipt of price.

